# Delineating family needs in the transition from hospital to home for children with medical complexity: part 2, a phenomenological study

**DOI:** 10.1186/s13023-023-02747-w

**Published:** 2023-12-12

**Authors:** L. van de Riet, M. W. Alsem, R. S. I. Beijneveld, J. B. M. van Woensel, C. D. van Karnebeek

**Affiliations:** 1 Department of Pediatric Intensive Care, Amsterdam UMC, Meibergdreef 9, Amsterdam, The Netherlands; 2 Amsterdam Reproduction & Development Research Institute, Amsterdam, The Netherlands; 3 On Behalf of the Transitional Care Unit Consortium, Amsterdam, The Netherlands; 4 Department of Rehabilitation, Amsterdam Movement Sciences, Amsterdam UMC, Meibergdreef 9, Amsterdam, The Netherlands; 5 Emma Center for Personalized Medicine, Departments of Pediatrics and Human Genetics, Amsterdam Gastro-Enterology Endocrinology and Metabolism, Amsterdam UMC, Meibergdreef 9, Amsterdam, The Netherlands

**Keywords:** Children with medical complexity, Hospital-to-home transition, Care pathway, Parental needs, Needs-oriented interventions, Phenomenology, Inductive thematic analysis, Qualitative research

## Abstract

**Background:**

A systematic literature review on the transition from hospital-to-home (H2H) of families with a child with medical complexity (CMC), resulted in nine overarching themes. These demonstrated common needs and experiences despite the widely differing CMC diagnoses and family characteristics. However, none of the reported studies was conducted in the Netherlands, which hampers the creation of a tailored H2H care pathway, deemed essential for our recently established Transitional Care Unit in the Netherlands: the *‘Jeroen Pit Huis’*. Therefore, the aim of this study was to gain a deeper understanding of the needs and experiences of Dutch CMC parents on H2H transition and integrate these insights with the literature review into an evidence-based H2H care pathway for CMC and their families.

**Methods:**

A descriptive phenomenological approach was applied. Heterogeneous purposeful sampling methods were used to recruit participants according to the following criteria: parents of CMC from various regions in the Netherlands, who spoke Dutch fluently and who had been discharged home from a tertiary hospital within the previous five years. Semi-structured, open-ended interviews were conducted via video call by two researchers, who transcribed the audio recordings verbatim. Thematic analysis methods were used to identify emerging themes from the individual transcripts, involving a third and fourth researcher to reach consensus.

**Results:**

Between March and August 2021, 14 mothers and 7 fathers participated in 14 interviews. They elaborated on the H2H transition of 14 CMC with a wide range of underlying diseases: 7 male, 7 female, aged 6 months to 10 years. Eight overarching themes, consistent with the results of the systematic review, represent CMC parental needs and experiences during the H2H process in the Netherlands: (1) autonomy, (2) division of tasks and roles, (3) family emotions, (4) impact on family life, (5) communication, (6) coordination of care, (7) support system and (8) adaptation.

**Conclusions:**

The H2H needs and experiences reported by the CMC families in this study align with the results of our systematic review. The H2H transition process is not linear but continuous, and should extend beyond the specific medical needs of the CMC to holistic care for the family as a whole. The overarching care needs and experiences, expressed by all CMC families, regardless of underlying symptoms and diagnoses, inform the H2H care pathway and its future evaluation. Our studies highlight the necessity to focus on the family needs rather than on the specific illness of the child, as well as the value of our interdisciplinary care team partnering with parents in the *‘Jeroen Pit Huis’* towards a safe and sustainable transition home.

**Supplementary Information:**

The online version contains supplementary material available at 10.1186/s13023-023-02747-w.

## Introduction

The number of children in chronic need of medical support has steadily increased in recent decades [1]. This can be attributed to a number of factors including improved outcomes after critical illness, better treatment options and the adaptation of medical devices for home use [2–5]. These chronically supported children, often referred to as children with medical complexity (CMC) [6], make extensive use of complex medical care through the involvement of many healthcare professionals. Living at home has been associated with several positive psychological, emotional and social benefits for both child and family and it is therefore desirable that children spend as little time as possible in the hospital [7–10]. However, the transition of care from hospital to home (H2H) poses many challenges for families of CMC, whose lives are disrupted by the complexity of the situation [9, 11, 12]. This often results in prolonged and recurrent hospital admissions and frequent emergency room visits after discharge [13, 14], impacting healthcare systems worldwide [3, 15–19].

The lack of a care pathway to support CMC families is an important gap in current H2H transition care, as highlighted by Brenner et al. [20]. To improve on this, their research focused on the core principles for effective and personalized CMC care. To create a tailored care pathway to improve the H2H transition for parents of CMC, involving them in the process is essential to identify their needs and barriers. Several studies have explored the H2H transition for CMC families [21–29], yet a clear overview of parental needs and experiences during this transition is lacking. Therefore, in preparation for this study, we conducted a systematic review to summarize the results of all qualitative studies focusing on H2H transition for CMC families. This resulted in nine overarching themes on which intervention programs could focus: (1) parental empowerment: shifting from care recipient to caregiver (2) coordination of care (3) communication and information (4) training skills (5) preparation for discharge (6) access to resources and support system (7) emotional experiences: fatigue, fear, isolation, and guilt (8) parent-professional relationship and (9) changing perspective: finding new routines.

While this systematic review provided us with valuable insights into the H2H transition as experienced by parents all over the world, the applicability of the results was hampered by the following factors. First, the methodological quality of the included studies varied, which may cloud the interpretation of the results. Second, the number of mothers included in the systematic review was disproportionally high. Finally, national and cultural differences may exist, yet none of the included studies has been conducted in the Netherlands. Knowing whether the same themes apply to the Dutch CMC population may give us insights in the H2H process in the Netherlands where we have recently established the *‘Jeroen Pit Huis’* as Transitional Care Unit to improve H2H care. Therefore, the aim of this study was to gain a deeper understanding of the needs and experiences of Dutch CMC parents on H2H transition and integrate these insights with the results of the literature review into an evidence-based H2H care pathway, tailored to the needs of CMC and their families.

## Methods

### Design

Since the aim of this study was to understand a particular phenomenon (i.e. parental experiences with H2H transition for CMC and their families) from the perspective of those experiencing it (i.e. the parents), a descriptive phenomenological approach was chosen [30–33]. Parents of CMC all experience H2H transition differently and therefore form unique opinions about the nature of H2H transition, but at the same time by studying them in depth, similarities can be revealed that help in the development of more tailored, family-centred interventions.

### Selection of participants

Participants were recruited using heterogeneous purposeful sampling. This way, a maximum variation sample was selected to ensure that the study contained rich and diverse data sources and that we captured a broad parental perspective. This involved identifying and selecting individuals from various regions in the Netherlands, who had considerable experience with the phenomenon of interest [34]. A letter explaining the study was distributed among potentially eligible parents of CMC by their clinical doctors. Once parents considered participating in the study, they were contacted by the researchers (LR and RB). Based on previous research, a sample size of approximately ten interviews was considered sufficient [35]. However, recruitment of eligible participants continued until no new experiences emerged from the interviews. To make sure that information saturation was truly achieved, an iterative process of collecting new data through interviews and simultaneous analysis of the data, continued until no more new insights could be added to the results.

### Inclusion and exclusion criteria

CMC was defined as individuals with: high family-identified needs; one or more complex chronic disease necessitating specialized care; functional disabilities; and high health care utilization [6]. To be included in the study parents had to be fluent in Dutch. In addition, they had to have experienced a hospital to home discharge after first HC/ICU admission within the previous five years.

### Research ethics

The study protocol W19_459#19.532 was approved by the Medical Ethics Review Committee of the Academic Medical Center in Amsterdam, The Netherlands. The participants were informed that they had the opportunity to withdraw from the study at any time.

### Data collection

Semi-structured, open-ended interviews were conducted between March and August 2021. Due to Covid-19 regulations the interviews were conducted via video call using Microsoft Teams (version 4.16.1). Prior to the interview, parents completed a questionnaire containing demographics and information about their child’s health status. The interviews were a combined endeavour by LR, medical doctor and PhD student working in paediatrics and RB, medical research student. The roles of lead interviewer and observer/support interviewer were alternated per interview. The lead interviewer presented herself, introduced the support interviewer and asked participants to elaborate on their experiences with the phenomenon [35].

Before the interview started, all participants verbally consented to have the interview audiotaped. To ensure consistency, a clear interview guide was defined [see Additional file 2]. The same questions were asked in all subsequent interviews, but the order of the topics was amended and guided by the parents’ answers. When needed, additional questions were added to provide clarification and a deeper understanding of the phenomenon. This semi-structured way of interviewing created space for individual stories and experiences. At the end of each interview that lasted an average of 60 min, the lead interviewer summarised the main points and offered the participants the opportunity to supplement new information or ask additional questions to the research team.

### Data analysis

Interviews were thematically analysed to identify emergent themes. The analysis was grounded in the procedures developed by Straus and Corbin, which involved *open coding*, *axial coding* and *selective coding *[36, 37]. MaxQDA 2022 (VERBI software, 2021) was used as the main analytical tool. First, the audio tapes were transcribed verbatim by the same researchers who conducted the interviews (LR and RB). Subsequently, the transcripts were cut into *units of meaning*: smaller fragments that described an experience or need, as part of the open coding process. These units of meaning were checked by the other team members (LR, RB, MA, CK) before categories were formed during axial coding. Consensus had to be reached among all team members before the final step, selective coding, summarized the core concepts into overarching themes.

### Research quality

Accuracy of the data was established through the verbatim transcripts and spot checking of transcripts by LR and RB. To ensure reliability and rigor of the data collection and analysis process, the full team (LR, RB, MA, CK) reviewed all stages of the coding process in regular meetings until consensus was reached on the final themes. Ideas were discussed and compared between the researchers for credibility [38]. Finally, to minimize potential researcher bias and increase accuracy of data, the results were sent back to the participants to check whether they resonated with their experiences [39].

## Results

### Sample

A total of 21 parents (7 fathers and 14 mothers) participated, representing the H2H transition of 14 children. During 7 interviews, both parents were present, the other 7 were conducted only with the mother. All parents were together and—all families but one—lived together at home. Four families made the transition home via a specialized transitional care unit and 1 patient was first transferred to a nursing home before eventually transitioning home. All other cases returned home directly from the hospital, either from the paediatric intensive care unit or a general paediatric ward. The duration of admission before H2H transition varied between 5 weeks and 2 years. Two parent couples withdrew from the study, because the memories evoked too many emotions. Table 1 summarizes the demographics of the study population. Table 2 provides clinical data of the CMC participating in this study.

**Table 1:** Family demographics

Participants (n, %)	
Total cohort of parents	21 (100)
Male	7 (33.3)
Female	14 (66.7)
Mean age parents (y, range)	
Overall	38.2 (28–50)
Male	40.0 (34–50)
Female	37.4 (28–42)
Nationality (n, %)	
Dutch	18 (85.7)
Iraqi	2 (9.5)
Moroccan	1 (4.8)
Highest level of education parents (n, %)	
Secondary school	2 (9.5)
Intermediate vocational education	4 (19.1)
Higher vocational education	11 (52.3)
University level	4 (19.1)
Children with Medical Complexity (CMC) (n, %)	
Total cohort	14 (100)
Male	7 (50)
Female	7 (50)
Mean age CMC (y, range)	
Overall	4.2 (6m–10y)
Male	4.4 (1y–7y)
Female	3.9 (6m–10y)
Number of siblings (n, %)	
0	6 (42.9)
1	2 (14.3)
2	4 (28.6)
3	1 (7.1)
4	1 (7.1)
H2H transition mode (n, %)	
From hospital to home	9 (64.3)
Via transitional care unit	4 (28.6)
Via nursing home	1 (7.1)
Home care (n, %)	
Yes	10 (71.4)
No	4 (28.6)

**Table 2:** Clinical data on CMC participating in this study

Interview no.	Congenital or acquired disease	Clinical presentation and diagnosis (if known)	Technological support at discharge	CMC score*
1 (A)	Congenital	Suspected genetic syndrome (global developmental delay, microcephaly, epilepsy, pulmonary hypertension, mono-kidney)	Percutaneous endoscopic gastrostomy tube feeding	4
2 (B)	Congenital	Deletion of chromosome 8 and duplication of chromosome 7 (global developmental delay, congenital pelvis abnormality with micturition and defecation problems, pulmonary hypertension)	Pulse oximeter, intestinal washing, percutaneous endoscopic gastrostomy tube feeding	4
3 (C)	Acquired	Progressive intellectual and neurologic deterioration of unknown cause	Tracheostomy, 24/7 invasive mechanical ventilation	4
4 (D)	Congenital and acquired	Congenital arthrogryposis multiplex and acquired brain injury	percutaneous endoscopic gastrostomy, tracheal tube, night time mechanical ventilation	4
5 (E)	Congenital	Down syndrome (tracheomalacia of unknown cause, sleep apnea)	Non-invasive ventilation, pulse oximeter	4
6 (F)	Congenital	SNC2A mutation with severe developmental delay and epilepsy	Nasogastric tube feeding, oxygen	4
7 (G)	Congenital	Partial duplication chromosome 9q, deletion chromosome 7b. Marfan phenotype. Multiple dysmorphies (micrognathia, frontal bossing, macrodactyly). Digestive tract varices caused by portal hypertension due to vena porta thrombosis	Nasogastric tube feeding, pulse oximeter	4
8 (H)	Acquired	Acute flaccid myelitis at the age of 7 years, due to an Enterovirus D68 infection	Tracheostomy, 24/7 invasive mechanical ventilation	4
9 (I)	Congenital	Gastroschisis requiring multiple surgeries	None	4
10 (J)	Congenital	Diaphragmatic hernia	Tube feeding, oxygen, pulse oximeter	4
11 (K)	Congenital	Oesophageal atresia and tracheomalacia of unknown cause	Tube feeding, continuous positive airway pressure	4
12 (L)	Congenital	Diaphragm paralysis of unknown origin (respiratory failure)	Mechanical ventilation, tube feeding,	4
13 (M)	Congenital	Spinal Muscular Atrophy with Respiratory Distress type 1	Mechanical ventilation on demand, oxygen, tube feeding	4
14 (N)	Congenital	VACTERL association: vertebral anomalies/anorectal malformations/cardiovascular anomalies/tracheoesophageal fistula/renal anomalies/limb defects, bronchopulmonary dysplasia, epilepsy	Mechanical ventilation, oxygen, tube feeding	4

*CMC score based on CMC criteria as published by Cohen et.al [6]

### Main themes

The process of inductive thematic analysis resulted in eight overarching themes regarding H2H transition for CMC: (1) autonomy; (2) division of tasks and roles; (3) family emotions; (4) impact on family life; (5) communication; (6) coordination of care; (7) support system; (8) adaptation. Each theme is discussed below. An additional file shows a more detailed overview of the eight themes [see  Additional file 1: Table 3], in which citations from parents are used to illuminate their experiences.

#### Autonomy

Many parents felt helpless during their time in the hospital. Having to allow other people to take care of their child was perceived as hard. When they were finally able to participate in the care of their child, some parents felt that they could take back control. Life in the hospital was extremely disruptive and involved a lot of waiting. Waiting for professionals to visit the child—and not knowing when they would come—or waiting for home equipment to be ready before they could be discharged. Parents lacked structure in their days. They felt as if their lives had been taken over by the rhythm of the hospital. Once home their sense of autonomy was not fully restored. Most families were supported by home care nurses, at least part of the time. As much as this help was needed and appreciated, it also led to a loss of privacy and private time for the family.

#### Division of tasks and roles

Taking care of CMC is teamwork and demands a clear division of tasks between parents and professionals. Many parents stressed the importance of involving and listening to them from the start and setting expectations about care delivery to avoid misunderstandings. Parents were generally eager to participate in their child’s care, but did not want this to be taken for granted by professionals. Simply being asked to take on the challenging caregiving role, could prevent negative feelings such as frustration and anger. Many parents indicated that a special parents-professional relationship developed over time, which created a sense of familiarity and trust. On a personal level, parents struggled with fulfilling a dual role: being both parent and caregiver. It was challenging to separate the two when parents felt compelled to take on a nursing role when they just wanted to be a parent to their child. All parents took on roles differently, as character differences between parents are magnified by the extreme circumstances.

#### Family emotions

Having a CMC and dealing with everything related to CMC care, evoked all kinds of emotions in parents. For most parents, their child was unexpectedly seriously ill, and a period of respite was suggested by them. This could allow them to catch their breath before being asked to take on an active caregiving role. To deal with all their negative and anxious feelings, parents emphasized the importance of a positive attitude of professionals, which gave them hope. Staying in the hospital for a long time, especially in the ICU, was traumatic. It was upsetting for parents to talk about all the grief they had experienced from other families in the ward. Several parents struggled with feelings of guilt, for example when their child survived, but other children in the ward did not, or when they had the feeling that they were unjustly occupying an ICU bed, awaiting the H2H transition. When they were finally able to go home as a family, parents encountered new obstacles, often related to arranging home care and medical equipment, or coping with an environment that underestimated their complex situation. These disappointing experiences sometimes resulted in a combative attitude and a strong desire to improve the system. At the same time, many parents trivialized the severity of their situation as a coping mechanism. Parents struggled with recurrent feelings of bereavement. Many of them had to deal with the near death of their child. After an initial relief when the child survived, continuing feelings of loss arose as well: loss of something that could have been, sadness when confronted with their child’s limitations. Ultimately accepting their new situation was an important part of the emotional process that the parents went through.

#### Impact on family life

Living with a CMC has a major impact on family life. In order to meet the fulltime care for their child, parents identified other aspects of their lives that also needed attention. For example, the importance of self-care was emphasized. Occasionally leaving the ward during the hospital stay of their child was necessary to cope with the intensity of their situation. Many parents wanted to lead a life of their own and to feel part of society. Being surrounded by people other than their immediate family, for example at work, strengthened their sense of identity. It was however a challenge for parents to balance their career and care role. Work made them feel happy and provided structure. At the same time, demanding jobs could not always be combined with CMC care, increasing parental stress and causing many parents to quit their jobs. Parents felt a strong need for family cohesion. During long hospitalizations, it was difficult for them to split up the family and for other relatives to not see each other for long periods of time. It was challenging for parents to divide their attention between the sick child and other siblings, whose daily rhythm was repeatedly disrupted as well. Ongoing care for CMC changed relationships, both between parents—who felt more like a manager than a partner—and between the parents and those around them. Friends and colleagues who struggled to understand their situation made parents feel disconnected, resulting in social isolation.

#### Communication

Communication is an important aspect of CMC care, and specific elements can make a big difference in parents’ perception of H2H transition. For example, many parents emphasized the importance of clear and personalized information about their child’s medical condition, without the use of medical jargon or information overload. In addition, one mother suggested that less assertive parents who were less likely to verbally express themselves, should not be overlooked. Openness about what to expect, both about their child’s prognosis and about their own future, was also mentioned as important. When parental expectations were not properly managed, it could lead to misconceptions and unexpected setbacks. During conversations about their child’s care and future prospects, parents wanted to be seen as equal partners. If the situation allowed it, they appreciated being informed by professionals before changing the treatment plan. Maintaining communication with their environment could be an additional stressor for parents. Some suggestions were made by parents to alleviate this burden, such as writing a blog or a regular update through a designated friend or family member.

#### Coordination of care

Coordinating care for CMC was perceived as difficult, especially after hospital discharge. Assistance from a case manager to navigate the range of institutions parents had to deal with, was found to be helpful. Furthermore, an overview of available services was suggested as a useful, practical tool to direct parents in the right direction. Another important aspect of care coordination was continuity of care. Seeing familiar faces in case of a readmission was reassuring and gave them a sense of calm. Some parents mentioned a lack of access to medical information across healthcare institutions. This resulted in both explaining their specific situation over and over, but more importantly, could lead to dangerous situations when healthcare professionals did not have access to their child’s medical record in an acute situation. Parents expected a certain level of expertise from professionals when they needed help coordinating different aspects of CMC care, unfortunately this was not always the case. While parents understood that the magnitude and complexity of healthcare initiatives could be overwhelming for professionals, as it was for them, they were often disappointed by a lack of knowledge and collaboration between different healthcare institutions.

#### Support system

In order to bare the challenging CMC care both during hospital stay and at home, parents mentioned the importance of a solid support system. Most parents were offered psychological support during a lengthy hospital admission. Although this was appreciated, a number of parents also indicated that timing is relevant since they were not open to it during the hectic first period. Some of them picked up on this later, others found emotional support elsewhere. For example, by talking to family and friends. In addition, peer support through the experiences from other CMC families provided comfort and a sense of familiarity. Connecting with other families facing similar challenges instilled their confidence. Tips and tricks from other CMC families were perceived as useful as well, as their experiences provided them with more ‘out of the box’ solutions to everyday problems. The use of social media could be an accessible way of connecting to other families. Some parents found support in their religion. On a more practical level, most parents received support from home nurses on a daily or weekly basis. They helped parents improve their nursing skills and took away some of the responsibility associated with providing CMC care at home. Most parents did not ask their friends and family to perform specific nursing tasks, preferring that their child had a normal relationship with their relatives. On the other hand, help with household chores or taking care of siblings during their absence was highly appreciated. Finally, parents emphasized the added value of respite care and school, which could ease the constant burden on the family and gave parents time to spend with siblings or themselves.

#### Adaptation

The H2H transition for CMC was often perceived as a lengthy process that families had to go through, ultimately resulting in adapting to new routines. Apart from time and perseverance, that were needed to adapt, parents also mentioned a number of practical steps that helped them do so. For example, they emphasized the added value of practicing before discharge. By taking full responsibility for their child’s care, while having the safe hospital environment to fall back on when needed, they realized what they were capable of, but also became aware early on of things they did not know yet. In addition, parents suggested a step-by-step care plan and a practical discharge checklist for discharge. Creating a contingency plan to be used in an emergency at home, gave them the confidence they needed to take their child home. Apart from taking these practical steps, parents also went through an emotional process. By constantly dealing with uncertainty, which could be challenging at times but also strengthened their flexibility and self-efficacy, parents eventually became expert caregivers.

## Discussion

The purpose of this phenomenological study was to engage Dutch parents of CMC in a dialogue to gain a deeper understanding of their needs and experiences on the H2H transition. An appropriate understanding of these needs is necessary for healthcare professionals to develop a care pathway, which supports CMC and their parents more needs-oriented and thus facilitate a more sustainable transition home. The needs and experiences of our Dutch CMC families consistently centred around eight themes: (1) autonomy, (2) division of tasks and roles, (3) family emotions, (4) impact on family life, (5) communication, (6) coordination of care, (7) support system and (8) adaptation. Although each of our CMC families is unique, our study shows that a variety of parents share similar H2H transition needs and experiences that can be summarized into these overarching themes. Our study revealed similar themes to previously published studies, which are included in our systematic review [24, 40, 41]. This not only supports the trustworthiness of our findings, but also strengthens the idea that a H2H transition care pathway should focus not merely on disease-specific medical issues and disease-specific nursing skills, but that a holistic approach that addresses all domains of care is needed. This was repeatedly emphasized by the parents.

Manhas et al. stated the following: ‘*Transition is not simply about moving a child home*’ [40]. This also resonated in our study population, as all parents emphasized that H2H transition was a demanding and continuous process; the initial transition from life as they knew it to an intensive period of being in a hospital, the numerous hospital transfers with their CMC, the multiple readmissions and finally, perhaps most importantly, the shift in parental responsibility and the adaptation to their new role. All parents expressed their willingness to become their child’s caregivers. They reported that the support and trust of healthcare professionals enabled them to build confidence in their own caretaking capabilities. The importance of professional support in learning (nursing) skills was also emphasized by Murdoch et al. [42]. Over time, however, parents became medical experts on their child’s health. This is important to keep in mind, as it is still common practice for healthcare professionals to take the leading role in decision making, planning or care delivery when a child is readmitted to the hospital [43]. Acknowledgement of parental expertise during the often frequent readmissions, should be embedded in CMC care.

Parents repeatedly expressed their frustration with the complexity of the regulations surrounding the transition home. While some parents needed more time to feel ready at the time of discharge, the majority expressed the counter-perspective of being able to be discharged earlier if logistical problems had not delayed this. Parents’ frustration with the transition process is well known, with common problems such as difficult financial arrangements, finding suitable home care nurses and dealing with many different agencies without a central point of contact [44, 45]. The introduction of a case manager, someone who parents can turn to with all their general and logistical questions, could address this issue. Previous research showed that the introduction of this role reduced parents’ time spent coordinating care for their child, ultimately resulting in more quality time with their child and time for self-care [46].

As our study is the first to focus on the experiences of CMC families on H2H transition in the Netherlands, it adds new information to the existing literature. For example, Berman et al. and Umberger et al. concluded in their studies that parents struggled to be involved in decision making [41, 47]. In contrast, the parents in our study reported that they felt they were being listened to and that their opinion could influence the care plan. Although speculative, the high valued shared decision making in the Netherlands may have contributed to this different perception. Our research demonstrated, consistent with the previous literature, the importance of an explicit, straightforward exchange of information in a respectful manner [48, 49].

Parents were grateful when healthcare professionals were aware of their psychological and mental well-being. As one parent put it: “We thought the care would be focused on the medical, the clinical part, but it is much more than that. It is not only taking care of our daughter, but also taking care of us as parents.” This is an important finding, as it highlights the importance of holistic care. More traditional care standards focus mainly on disease management [50]. Our results indicate that CMC care encompasses many different aspects of care delivery, of which the disease itself—and the medical and nursing skills and tools that accompany that disease, are merely a part of the total care ‘experience’. In addition, many CMC have multiple diagnoses or none at all. In this light, it makes more sense to develop H2H transition care programs for CMC and their families based on their needs, rather than their diseases.

Four of the interviewed parents were very positive about their stay in a transitional care unit, where their expectations regarding the quality and consistency of their training to care for their child was exceeded. Previous studies reported higher readmission rates for children whose parents did not feel prepared to manage their child’s health after discharge [51]. This supports the fact that family-tailored discharge education is associated with improved patient outcomes and reductions in potentially avoidable healthcare utilization [52]. The overarching themes will be implemented in the transitional care path that is being carried out at a newly opened transitional care unit in the Netherlands [53, 54]. Future studies will evaluate this (preliminary) framework and test its accuracy in improving H2H transition care.

One of the main strengths of our study was the participation of a relatively high number of fathers, resulting in a deeper understanding of the phenomenon. Unlike many other studies, where mothers are often the sole representatives of the family [40, 42, 55–57]], fathers were present in half of our interviews. Although we attempted to have a maximum variation sample, including only Dutch-speaking families may have prevented us from exploring the experiences of parents from different backgrounds living in the Netherlands, which could have limited the generalizability of our findings. On the other hand, there were several factors that support the heterogeneity of our participants and strengthen the applicability of our results. First, the parents in our study varied widely in age and level of education. Second, our CMC families were spread across a significant part of the Netherlands, both in rural and urban areas. Third, the participants represented both families who had recently made the transition home and families who had experienced it up to five years ago. Fourth, our study reflects the experience of parents who made the transition home directly from the hospital and of parents who went through a transitional care unit or a nursing home. Fifth, not only the participating parents, but also the CMC, represent a diverse group of children suffering from a wide range of rare diseases.

Due to Covid-19 restrictions, interviews could not be conducted face-to-face, but via video call. Some qualitative methodologists may argue that this approach hinders the connection with participants and could therefore lead to less comprehensive data [58, 59]. However, this was not our experience. A connection quickly developed between parents and researchers during the video call, which led to a relationship of trust that allowed for the interview to deepen and for the interviewers to respond accurately to non-verbal body language. Moreover, a major advantage of this approach was also the time efficiency. Because CMC parents’ time is often limited, this approach may have increased their willingness to participate. A study by Krouwel et al. showed similar results between face-to-face interviews and video calls, the latter also being more time and cost-effective [60].

In transcendental phenomenology, the researchers must be vigilant in the self-reflective process of ‘bracketing’, in which they set aside prior knowledge, assumptions, and experiences about the phenomenon during interviews, to avoid biasing their individual subjectivity in data analysis and interpretations [61]. To minimize this limitation, we decided not to use the preliminary results of our systematic review when conducting the interviews. Instead, we used inductive thematic analysis methods to analyze our interview transcripts. Although we could not completely erase our prior knowledge on the topic, we believe that our methods strengthen the validity of our results.

## Conclusion and implications/future perspectives

The H2H needs and experiences reported by the Dutch CMC families in this study align with the results of our systematic review. The H2H transition process is not linear but continuous, and should extend beyond the specific medical needs of the CMC to holistic care for the family as a whole. The overarching care needs and experiences, expressed by all CMC families, regardless of underlying symptoms and diagnoses, inform the H2H care pathway and its future evaluation. Our studies emphasize the necessity to focus on the similarities within CMC families rather than on the specific illness of the child. Additionally, our results underline the value of an interdisciplinary care team partnering with parents towards a safe and sustainable transition home.

Further research is needed to support these findings and achieve changes in public policies. We are taking the first steps in this direction in the recently established the *‘Jeroen Pit Huis’,* the first Transitional Care Unit in the Netherlands (https://hetjeroenpithuis.nl). By collaborating within a national research consortium (https://www.tcuconsortium.nl), we aim to assess the generalizability of our results to clinical practice and to evaluate the H2H care pathway. Subsequently, the transitional experiences of non-Dutch speaking families need to be examined, because these parents might have different perspectives and therefore advice and support can be tailored to their individual needs as well.

### Supplementary Information

**Additional file 1: Table 3.** Final eight themes representing the parental H2H perspective, constructed from categories and illustrated through citations.

**Additional file 2.** Interview guide.

## Data Availability

The dataset supporting the conclusions of this article is partly included in Additional file 1: Table 3. Upon request, a complete dataset in the form of a MaxQDA file including the full coding file, can be shared, while respecting the confidentiality of the participants.
