# Supplementary material for: Delineating family needs in the transition from hospital to home for children with medical complexity: part 2, a phenomenological study
Source: Orphanet J Rare Dis. 2023 Dec 12;18:387. doi: 10.1186/s13023-023-02747-w (PMC10714565; doi:10.1186/s13023-023-02747-w)
Supplement: Supplementary file 1 — Additional file 1: Table 3. Final eight themes representing the parental H2H perspective, constructed from categories and illustrated through citations. [file 13023_2023_2747_MOESM1_ESM.pdf]

Additional file 1

Table 3: *Final eight themes representing the parental H2H perspective, constructed from categories and illustrated through citations*

Themes	Categories	Citations
Autonomy	Control (n)	"It was…she must have been six weeks old, the first thing I could do apart from changing a diaper, to help taking care of my child. It felt great, as if I could take back control." – *K*
	Structure (n)	"You don't know exactly when the doctors will pay you a visit. You are waiting for people to come by. You can't plan your own day. It is exhausting." – *E*
	Privacy (e)	"His grandmothers are around three times a week as well. It may sound weird, but I am never alone with him. There is always someone present to help, because his care is too complex. I find that very hard, I have no privacy anymore." – *N*
Division of tasks and roles	Clear division of tasks (n)	"They [the nurses] have to be aware of what parents want. It is very important to come to an agreement together. 'So, your child is being admitted. We expect you to be present so many times' or 'you have to be here all day'. It is important to talk about these things together from the start." – *B*
	Be involved and listened to (n)	"We were involved in the decision-making process and we were regularly asked for our opinion. We are not healthcare specialists, but it felt good to be involved." – *J*
	Being asked to take on caregiving role (n)	"I actually missed someone asking us if we were ready and willing to take our child back home with all the complex care. It was just assumed."- *D*
	Special parent-professional relationship (e)	"She had stayed in the hospital for a long time, they [professionals] started to feel like family ha-ha! Maybe she was special, she had been there for so long, all the nurses knew her, knew us." – *L*
	Dual role: being both parent and caregiver (e)	"You should be able to just be a parent." – *A*
	Parents take on roles differently (e)	"Because she needs complex and intensive care, I feel that the different character traits of the parents are magnified." – *A*
Family emotions	Respite (n)	"Some sort of mental preparation, almost like a retreat. 'Take your time to process things, just be with your child'. Someone to take care of you, allow you to catch your breath."– *D*
	Positivity (n)	"Stay positive. Doctors and nurses have to stay positive. It is key!"– *F*
	Traumatic (e)	"You are surrounded by so much sadness. You see a family getting together in the common room, because their child has just died. That's really intense, there's so much grief."– *H*

	Guilt (e)	"These other people had a two-year old son who died in the ICU. It was hell. We were in there together and I felt guilty that our son had survived."– *F*
	Combative (e)	"All these protocols and conditions, so many conditions, it is unreal! If you don't follow their rules…"– *M*
	Coping (e)	"You're starting to make different choices, your priorities shift. It has a major impact on your life, yes."– *A*
	Bereavement (e)	"Life, our reality, has completely changed. Telling you this is really hard."– *L*
	Acceptance (e)	"That is her, with the tube. It is extremely important for you to accept your child as it is."– *G*
Impact on family life	Self-care (n)	"Every now and then you have to leave the ICU, for yourself as well."– *H*
	Societal participation (n)	"You also want to lead a normal life, even though you have a child who is severely ill."– *F*
	Balance career and caregiving role (n)	"It is a difficult balance. Work can be demanding, but it also gives you energy."– *A*
	Family cohesion (n)	"The thing we found extremely hard was that our other children did not see him for over eight weeks after he was born."– *I*
	Changing relationships (e)	"It is a recipe for disaster, for your relationship as well. You are constantly managing everything, you feel more like business partners. And of course you are parents."– *D*
	Social isolation (e)	"So at some point you want to start living again, but you notice that you're somewhat isolated. Also at work, you don't connect with other parents. Their children go to primary school and that is great. They hang out with other parents so that their children can play together. Good for them. We can't do those things, no we can't. "– *A*
	Parents as manager(s) (e)	"We run our own healthcare company ha-ha. At night we do all the paperwork. It is horrible, it is not by choice. We are capable to do it, but it is out of necessity."– *D*
Communication	Clear and personalized information (n)	"They told us straight up that she was severely ill, but – luckily – she was not going to die. That was a relief."– *H*
	Openness (n)	"Parents should know what to expect. You are not just going to learn new (nursing) skills, it is much more than that. Some parents may experience less extreme situations, but all of them will run a medical business, at least part-time."– *D*
	Equal partnership (n)	"The communication here was really good. They involved us in everything, really listened to us. I like that very much."– *N*

	Communication with environment (n)	“We wrote a blog to inform everyone around us. It worked on two levels, it helped me to deal with it (by writing about it) and it helped us to inform everyone at once.” – *I*
Coordination of care	Navigation (n)	“Once you know that a child is going to require complex, intensive care, someone should join (and support) a family. Someone to navigate that jungle. That’s what it is, a jungle.”– *A*
	Overview of available services (n)	“It would be really nice if you got some kind of toolbox. If that’s what you’re dealing with, you should go there.”– *D*
	Continuity (n)	“It is funny ha-ha, whenever we are readmitted to [department name], there are three nurses arguing about who gets to take care of her. It is nice to have familiar faces, they know you.”– *G*<br><br>“It is really nice to have someone call you a week after you’re discharged home to ask how you are doing. And not just to discuss the medical issues.”– *E*
	Access to medical information (n)	“That was our biggest frustration. Each time we have to go to the emergency room, she needs [name of a drug] and it has to be prepared in a certain way. But every time we’re there, this is not done correctly! You wonder why you bother to tell them? It is written down in her file.. It frustrates me.”– *G*
	Professional expertise (n)	“They should summarize all these initiatives. It might be difficult, but… also for healthcare professionals, when they face a situation like ours, that they know where to go for information. It’s a jungle, for us, but for healthcare professionals as well. There are so many initiatives.”– *A*
Support system	**Emotional support**	**Emotional support**
	Psychological support (n)	“From the start, they offered psychological support. Someone would come over to talk to us. We needed that. We felt supported, we really appreciated that.”– *C*
	Talk to family and friends (n)	“Mostly for mental support. We have really close friends we can talk to.”– *G*<br><br>“I get along with our neighbour really well. If I wasn’t here with [name child], I would sometimes visit her to talk.”– *H*
	Experiences from other CMC families instils confidence (n)	“It is nice to have the option to talk to other parents. They recognize what you’re going through, unlike most people that surround you, since they simply don’t go through the same things as you do.”– *I*
	Religion (e)	“I believe in God, I feel that my faith has given me a lot of support.”– *I*
	**Practical support**	**Practical support**
	Home nurses (n)	“They [the home nurses] allow us to do other things, like grocery shopping, taking a nap or just relax for a while.” – *G*

	Help from family and friends at home (n)	“Sometimes our friends brought meals over.” – *D*
	Tips and tricks from other CMC families (n)	“We don’t have all the information, so I asked other parents how they are handling everything.” – *K*
	Respite care and school (n)	“She will go to medical day care. It is also possible for her to stay the night, if that is what we need sometimes.” – *K*
Adaptation	Requires time and perseverance (n)	“It definitely depends on your own character as well. I like to find solutions. If something doesn’t work, we try it another way, but it takes a long time and you need a lot of energy and perseverance.”– *G*
	Practice before discharge (n)	“It is good to practice on your own in the hospital for a full day. The nurses should not do anything and say to us: ‘show us what you’re doing’.”– *B*
	Discharge checklist (n)	“I think it would be very helpful for parents, even if it is just a simple practical checklist that you go through together before discharge. Not just ask: ‘I think you have everything?’”– *N*
	Care plan (n)	“They should ask you prior to discharge: ‘What needs to be done? What do we need to arrange for you? A step-by-step care plan, not just the medical issues.”– *B*
	Contingency plan (n)	“We went home with a contingency plan. When do you call the ICU, when do you contact your GP or when do you call an ambulance? We also took a first aid course before we were allowed to go home.”– *H*
	Deal with uncertainty (e)	“We had to adapt to a new way of life. We had to learn to deal with a care-intensive child, a future that was uncertain.”– *D*<br><br>“That was very hard. Every time you thought you’d had the worst, a new problem arose.”– *G*
	Self-efficacy (e)	“At some point, I think he was a year and a half, I said ‘I know everything I need to know, I know how to act’. I built up the confidence and I wanted to bring him home.”– *M*<br><br>“It is such a relief when you dare to do everything yourself and that you *can* do everything yourself.”– *G*
	New routines (e)	“I just really wanted things to be alright for all my children, all three of them. No matter how well you try, you can never give them the time you want.”– *K*

H2H = Hospital-to-home; n = need; e= experience
