# Supplementary material for: Delineating family needs in the transition from hospital to home for children with medical complexity: part 2, a phenomenological study
Source: Orphanet J Rare Dis. 2023 Dec 12;18:387. doi: 10.1186/s13023-023-02747-w (PMC10714565; doi:10.1186/s13023-023-02747-w)
Supplement: Supplementary file 2 — Additional file 2. Interview guide. [file 13023_2023_2747_MOESM2_ESM.pdf]

Delineating family needs in the transition from hospital to home for children with medical complexity: part 2, a phenomenological study

Interview guide

Introduction of the topic, the expected duration of the interview and explanation of the semi-structured interview method. Acknowledgement of the voluntary participation of parents and informed consent to the recording of the interview.

Part 1: Hospital stay and working towards discharge

1. **Could you tell us a bit more about your child's hospital admission?**
   Supportive questions which could be used to clarify the main question.
   - How did your child end up in the hospital?
   - What was the duration of the hospital admission?
   - Was this the first time that your child (or one of your children) was admitted to a hospital?
2. **Could you tell us a bit more about the last part of the hospital admission, during which you anticipated the transition from the hospital to home with your child?**
   Supportive questions which could be used to clarify the main question.
   - Was it a gradual process working towards hospital discharge, or was it an abrupt moment? Who decided when you were ready to transition home?
   - Was it clear to you how and when you would be discharged? Were there, for example, clear steps to follow or was there a discharge checklist? If not, would you have liked some sort of discharge guideline?
   - What was your role in the discharge process? Where you in control or did healthcare professionals decide this for you? Were you involved in the decision making? How did that make you feel?
   - Where there other paramedics involved, such as:
     - Social work
     - Psychologist
     - Pedagogical support
     - Physical therapist
     - Occupational therapist
     - Rehabilitation doctor

3. **(If applicable) How did you experience the transition from hospital towards another care facility, such as a nursing home or a rehabilitation center?**

   Supportive questions which could be used to clarify the main question.
   - Who decided where your child would transition to?
   - Did the healthcare professionals talk to you about different options? If so, did you feel competent/able to make that decision yourself?
   - Did you feel supported making this decision?

Part 2.1: First period at home – general experience

4. **If you look back at the first period at home after you were discharged from the hospital, what is the first thing that comes to mind? This can be both a positive and a negative experience. Could you elaborate a bit more on these first weeks after discharge.**

   Supportive questions which could be used to clarify the main question.
   - Why was this such an important experience (both positive and/or negative)?
   - How did you cope with the situation?
   - What role did the healthcare professionals play in this phase?

Part 2.2: First period at home – specific issues

<u>Practical issues</u>

5. **Was it clear to you which practical matters had to be arranged for your child after discharge?**

   Supportive questions which could be used to clarify the main question.
   - You could think of (medical) equipment, adjustments to your home, arrangement of medication, transportation and financial support.
   - How did you experience the support of the hospital in this matter?
   - Could the hospital have supported you better? If so, do you have any suggestions?
   - Do you have home care and if so, how is this arranged?

<u>Information</u>

6. **Could you explain to us how you get information on all aspects of living at home with a child with a complex medical condition?**
   - Is this easily accessible to you?
7. **Was it clear to you how to respond in case of an emergency?**
   - Did you have a contingency plan at home?

Work and family

8. **How has caring for your complexly ill child affected your work and family life?**

   Supportive questions which could be used to clarify the main question.

   - Was it possible to continue working during this process? How did the contact you're your employer go?
   - Were there other siblings you had to take into account? If so, can you indicate how you arranged their care?
   - How was your contact with the school of your ill child, or if applicable, the school of other children in the family?

Support system

9. **A lengthy hospital admission is challenging in many ways, and has a major impact on the ill child and the parents, but also on other relatives, friends and family involved. Could you tell us a bit more about the way in which your child's illness and hospital admission(s) impacted your environment? Furthermore, how did you experience support from them?**

   Supportive questions which could be used to clarify the main question.

   - What was the impact on other family members, like siblings or grandparents?
   - Did you receive support from the people around you?
   - Did you receive support from healthcare professionals?
   - Did this change after you were discharged home?

Communication

10. **Could you tell us a bit more about the way in which healthcare professionals from different organizations communicated and collaborated?**

    Supportive questions which could be used to clarify the main question.

    - Was the general practitioner involved?
    - If so, at what time during the transition from hospital home, did he get involved?
    - Were there other healthcare professionals involved, who were not mentioned yet?
    - What was the communication like between all parties involved? What was your own role in this? How did you feel about that?

Part 3 – Needs and solutions of parents

Brief summary of what has been discussed.

11. **To finalize, looking back at the entire process of transitioning between the hospital and home with your ill child, what are the most important interventions/experiences that were helpful to you during this time?**

- Could you formulate a take home message for us?

12. What can we do to improve the transition from hospital to home for families like yours, who take care of a complex, chronically ill child?

- Could you formulate a take home message for us?

End of the interview

- Room for additional quesions or experiences from participants.
- Explanation (again) of the purpose of the research and the further processing of the data.
- Explanation about the member check.
